# Contributions of EspA Filaments and Curli Fimbriae in Cellular Adherence and Biofilm Formation of Enterohemorrhagic *Escherichia coli* O157:H7

**DOI:** 10.1371/journal.pone.0149745

**Published:** 2016-02-22

**Authors:** Vijay K. Sharma, Indira T. Kudva, Bradley L. Bearson, Judith A. Stasko

**Affiliations:** 1 Food Safety and Enteric Pathogens Research Unit, National Animal Disease Center, ARS-USDA, Ames, Iowa, United States of America; 2 Agroecosystems Management Research Unit, National Laboratory for Agriculture and the Environment, ARS-USDA, Ames, Iowa, United States of America; 3 Microscopy Services Laboratory, National Animal Disease Center, ARS-USDA, Ames, Iowa, United States of America; U. S. Salinity Lab, UNITED STATES

## Abstract

In *Escherichia coli* O157:H7 (O157), the filamentous structure of the type III secretion system is produced from the polymerization of the EspA protein. EspA filaments are essential for O157 adherence to epithelial cells. In previous studies, we demonstrated that O157 *hha* deletion mutants showed increased adherence to HEp-2 cells and produced abundant biofilms. Transcriptional analysis revealed increased expression of *espA* as well as the *csgA* gene, which encodes curli fimbriae that are essential for biofilm formation. In the present study, we constructed *hha espA*, *hha csgA*, and *hha csgA espA* deletion mutants to determine the relative importance of EspA and CsgA in O157 adherence to HEp-2 cells and biofilm formation. *In vitro* adherence assays, conducted at 37°C in a tissue culture medium containing 0.1% glucose, showed that HEp-2 cell adherence required EspA because *hha espA* and *hha csgA espA* mutants adhered to HEp-2 cells at higher levels only when complemented with an *espA*-expressing plasmid. Biofilm assays performed at 28°C in a medium lacking glucose showed dependency of biofilm formation on CsgA; however EspA was not produced under these conditions. Despite production of detectable levels of EspA at 37°C in media supplemented with 0.1% glucose, the biofilm formation occurred independent of EspA. These results indicate dependency of O157 adherence to epithelial cells on EspA filaments, while CsgA promoted biofilm formation under conditions mimicking those found in the environment (low temperature with nutrient limitations) and in the digestive tract of an host animal (higher temperature and low levels of glucose).

## Introduction

Enterohemorrhagic *E*. *coli* O157:H7 (O157) is an important foodborne pathogen of humans, causing symptoms ranging from watery diarrhea to hemorrhagic colitis and hemolytic uremic syndrome [1, 2]. O157 encodes a variety of cell surface structures that directly or indirectly promote its adherence to intestinal epithelial cells as well as cultured epithelial cells *in vitro* [3–5]. The EspA filaments are needle-like extensions of the type-three secretion system (T3SS) [6]. The T3SS spans both inner and outer bacterial cell membranes of O157 and about 20 genes encoded in the locus of enterocyte effacement (LEE) are required for the assembly of the T3SS [7–10]. The T3SS secretes virulence proteins called effectors that are delivered and injected into host epithelial cells through EspA filaments [8, 11, 12]. EspA, which is secreted by the T3SS, polymerizes at the tip of the needle of the T3SS, located in the outer membrane, to form hollow filaments measuring 12 nm wide and reaching a length of 260 nm or longer depending on the availability of the secreted EspA protein [6, 8, 13, 14]. Besides serving as a conduit for the translocation of effectors, EspA filaments also serve as adhesive elements and promote initial adherence of O157 to epithelial cells [15, 16]. LEE also encodes an effector protein called translocated-intimin receptor (TIR) that is secreted by the T3SS and translocated through EspA filaments into host epithelial cells [15–18]. TIR subsequently localizes in the epithelial cell’s cytoplasmic membrane to serve as a receptor for a LEE-encoded bacterial outer membrane adhesin, called intimin [17]. TIR-intimin interactions facilitate intimate adherence of O157 bacteria to cultured mammalian cells, intestinal epithelial cells of primary reservoir animals, such as cattle, and incidental human hosts [16, 19–25]. In O157-infected cattle and other ruminants, intimate adherence to mucosal tissues located proximal to the rectoanal junction leads to the formation of characteristic histopathology, termed attaching and effacing lesions [16, 19–22, 24–28]. A recent study has also demonstrated that EspA filaments are involved in biofilm formation by enteropathogenic *E*. *coli* [29].

In addition, the ability to adhere and colonize mammalian intestinal tissues by the LEE-encoded intimate adherence mechanism initiated by EspA-epithelial cell interactions, O157 isolates have been shown to adhere to animal tissues, plants, and abiotic materials, such as plastics, by employing unique sets of non-LEE encoded cell surface structures and proteins [30–38]. For example, adherence to abiotic materials, which generally begins by the cessation of the planktonic mode of existence, requires the expression of curli fimbriae [39–41]. Curli fimbriae are highly aggregative bacterial functional amyloids promoting initial irreversible bacterial adherence to abiotic surfaces as well as subsequent cell-cell interactions [42]. Curli fimbriae are also an important constituent of the extracellular matrix of mature biofilms [39–43]. Curli fimbriae, which range in size from 6 to 12 nm wide and 0.5 to 1 μm long, are composed primarily of the curlin protein encoded by *csgA* of the *csgBAC* operon, which is transcribed divergently from the *csgDEFG* operon [36]. The transcriptional regulator CsgD, encoded by *csgD*, is essential for the activation of these two operons [44, 45]. The *csgDEFG* operon also encodes proteins needed for the transport of CsgA and the nucleator protein CsgB across the bacterial outer membrane [46, 47]. Besides promoting survival of *E*. *coli* in the environment through biofilm formation, there is increasing evidence that curli fimbriae contribute to virulence and dissemination of *E*. *coli* in animals by enhancing bacterial interactions with a variety of host matrices and contact phase proteins [31, 36, 48]. Curli fimbriae have also been shown to promote bacterial interactions with cultured epithelial cells, enhance bacterial invasion in animal models, and contribute to severity of disease development in humans [32, 35, 49].

In previous studies, we demonstrated that *hha* is a negative transcriptional regulator of LEE, including LEE-encoded *espA*, and the operons *csgDEFG* and *csgBAC* that encode proteins mediating biosynthesis of curli fimbriae [33, 50]. Hha-mediated negative regulation of LEE and the two curli operons results from direct repression of genes encoding transcriptional regulators Ler and CsgD, which activate expression of genes encoded by LEE and the two curli opreons, respectively [33, 50]. Thus, a *hha* deletion in O157 allowed increased expression of LEE, enhanced secretion of effector proteins including EspA, and increased adherence of the *hha* mutant strain on cultured epithelial cells [50, 51]. Deletion of *hha* also resulted in the production of higher amounts of biofilm due to increased expression of *csgA* and other genes required for regulation and biosynthesis of curli fimbriae [33]. In the present study, we investigated whether increased expression of *espA* and *csgA* in the *hha* mutant strain of O157 contributed to increased adherence and biofilm formation independently or if both genes had incremental effects on these two processes.

## Materials and Methods

### Bacterial strains, culture media, and growth conditions

Bacterial strains and plasmids used in this study are listed in Table 1. All strains were derived from a streptomycin-resistant isolate of EHEC O157:H7 strain 86–24 originally linked to a foodborne disease outbreak in humans [52, 53]. Bacterial strains were cultivated in Luria-Bertani broth (LB) or LB agar supplemented with antibiotics as needed (streptomycin 100 mg per liter; kanamycin 50 mg per liter; and carbenicillin 100 mg per liter).

**Table 1:** Bacterial strains and plasmids^a^.

Strain or plasmid	Genotype and description	Source or reference
***E*. *coli* strains**		
86–24	*stx2*^+^ and streptomycin-resistant *E*. *coli* O157:H7	[52, 53]
6431	Δ*stx2* derivative of 86–24	[50]
6491	Δ*hha* mutant strain of 6431	[33]
6543	Δ*csgA* mutant of 6491	[33]
6550	Δ*espA* mutant of 6491	This study
6552	Δ*espA* mutant of 6543	This study
TOP 10	F^−^ *mcrA* Δ(*mrr-hsd*RMS-*mcr*BC) Φ80*lacZ*ΔM15 Δ*lac*X74 *rec*A1 *ara*D139 Δ(*ara*-*leu*)7697 *gal*U *gal*K *rpsL* (Str^R^) *end*A1 *nup*G	Life Technologies
**Plasmids**		
pCRXL	Cloning vector	Life Technologies
pACYC177	Low-copy cloning vector	New England Biolabs
pBluescript II SK	Cloning vector	Agilent Technologies
pSM694	0.8 kb α-complementation fragment of *lacZ* (isolated by PCR from pBluescript) cloned at *Bam*HI and *Bst*EII sites of pACYC177 for blue-white screening of bacterial colonies	This study
pAM450	Plasmid with a temperature-sensitive origin of replication	[50, 55]
pSM552	1.85 kb fragment carrying the 578 bp *espA* gene, 660 bp upstream, and 627 bp downstream of *espA*	This study
pSM556	pSM552 deleted of *espA*	This study
pSM601	0.86 kb fragment carrying the kanamycin resistance gene of the oBBI 92/93-neo cassette cloned at *Sal*I site located between upstream and downstream sequences of pSM552	This study
pSM615	2.2 kb *Xba*I fragment (carrying the kanamycin resistance gene flanked by the upstream sequence at the 5’ and the downstream sequence at the 3’ end) cloned at *Xba*I site of pAM450	This study
pSM706	pSM694 containing the *espA-* complementing DNA fragment isolated by PCR from 6431	This study
pSM708	pSM694 containing the *csgA-* complementing DNA fragment isolated by PCR from 6431	This study

^a^ Detailed descriptions of the construction of bacterial strains and plasmids listed are provided under material and methods.

### Determination of bacterial growth rates

Bacterial strains were grown overnight on a shaker (200 rpm) incubator at 37°C in LB broth containing carbenicillin (100 μg/ml). The overnight cultures were diluted 1:1000 and adjusted to equivalent optical density readings at 600 nm (OD_600_) in YESCA (0.1% yeast extract and 1% casamino acids) broth and Dulbecco’s Modified Eagle’s Medium containing 0.1% glucose (DMEM) (Life Technologies, Grand Island, NY). Both media also contained carbenicillin at 100 μg per ml. These diluted samples (400 μl) were inoculated into the wells of 120-well Honeycomb-2 plates. The plates were incubated at 37°C or 28°C in an automated growth curve reader programmed for continuous shaking and collecting OD_600_ at 30 min intervals (Growth Curves USA, Piscataway, NJ). The OD_600_ data were analyzed by using GraphPad Prism 6 software (GraphPad Software, Inc., La Jolla, CA). The generation times of bacterial strains were estimated from the exponential portions of the growth curves and equaled the time required for the doubling of OD_600_.

### Recombinant DNA procedures

The in-frame *espA* deletion mutants of EHEC O157:H7 strains 6491 (Δ*hha*) and 6543 (Δ*hha* Δ*csgA*) were constructed by using plasmid pSM552. Plasmid pSM552 was constructed by cloning a 1.85 kb fragment generated by PCR using primers VS585 and VS972 (Table 2) and genomic DNA of O157 strain 86–24. The 1.85 kb fragment contained 630 bp upstream of the *espA* ORF (Open Reading Frame), 578 bp of the *espA* ORF, and 660 bp downstream of the *espA* ORF. The PCR was carried out using FailSafe PCR Kit (Epicenter) according to the manufacturer’s instructions. The PCR fragment was purified by agarose gel electrophoresis. The DNA fragment was recovered from an agarose gel using a Gel Extraction Kit according to manufacturer’s instructions (Qiagen, Valencia, CA). The gel-extracted 1.85 kb fragment was ligated into pCRXL vector according to the manufacturer’s instructions (Invitrogen, Grand Island, NY). The ligated DNA was electroporated into *E*. *coli* TOP10 cells as described above. The recombinant plasmid pSM552 (pCRXL-1.85 kb fragment), thus constructed, was subjected to PCR using primers VS257 and VS941 to delete the 578 bp *espA* ORF. The *espA*-deleted PCR fragment of pSM552 was purified from agarose gel slices as described above and digested with *Sal*I to generate cohesive ends in *Sal*I restriction sites present in primers VS257 and VS941. The *Sal*I fragment was self-ligated and electroporated into an *E*. *coli* TOP10 bacterial host. The resultant recombinant plasmid (pSM556) was purified from a TOP10 isolate. The 0.86 kb kanamycin resistance fragment (*kan)* generated by PCR from oBBI 92/93-*neo* cassette [54] using primers VS777 and VS778 was cloned at the *Sal*I site generated by a deletion of the *espA* ORF in pSM556. The new *espA*-deleted plasmid (pSM601) was digested with *Xba*I to isolate a 2.2 kb DNA fragment containing 630 bp upstream of the *espA* ORF, 0.86 kb *kan*, and 660 bp downstream of the *espA* ORF. The 2.2 kb *Xba*I fragment was cloned at the *Xba*I site of plasmid pAM450, which encodes ampicillin resistance and a temperature-sensitive origin of replication [50, 55]. The new recombinant plasmid (pSM615) consisting of pAM450-2.2 kb fragment was electroporated into strains 6491 and 6543 for deleting the *espA* gene by using a previously described allelic replacement method [50]. The genomic DNA from kanamycin-resistant and ampicillin-sensitive mutants was screened by PCR using primers VS585 and VS972 (Table 2) to confirm that the *espA* gene was deleted and it was substituted by the 0.86 kb *kan* fragment. For complementation experiments, a 4.2 kb DNA fragment containing the *espA* gene was isolated by PCR from strain 86–24 using primers VS1085 and VS1086. Similarly, the 1.7 kb DNA fragment containing the *csgA* gene was isolated from parental strain 86–24 by PCR using primers VS1087 and VS1088. These individual fragments were purified from agarose gels as described above, digested with *Xba*I to produce cohesive 5’ and 3’ termini compatible for ligation into the *Xba*I site of pSM694, a low-copy derivative of pACYC177 (New England Biolabs, Ipswich, MA) carrying a 0.8 kb α-complementation fragment of β-galactosidase (Table 1). The ligated DNA mixtures were separately transformed by electroporation into *E*. *coli* TOP10 according to manufacturer’s instructions. The new recombinant plasmids generated by this ligation/electroporation were named pSM706 and pSM708. Plasmids pSM706 and pSM708 were electroporated into strains 6550 (Δ*hha* Δ*espA*) and 6552 (Δ*hha* Δ*csgA* Δ*espA*) to complement deleted *espA* and *csgA* functions, respectively. The empty vector pSM694 was also electroporated into the parental and all other strains so that these strains could be used as controls in subsequent experiments.

**Table 2:** Primers used for PCR.

Primer	Nucleotide sequence^a^	Location^b^ or reference
*E*. *coli* Primers^c^		
VS257_F_	CAGGTCGACCTATATACCTCTTGATAATTTTTC	4662701–4662724
VS585_F_	GCGTCTAGACATCGACTGCCGTTTGCAGTG	4661472–4661493
VS777_F_	GATCGTCGACCGATAGCTGAATGAGTGACGTGC	[54]
VS778_R_	GATCGTCGACGCATAGAGCAGTGACGTAGTCGC	[54]
VS941_R_	GATCGTCGACCCGGAGATAACTATGCTTAAC	4662122–4662101
VS972_R_	CAGTCTAGAAGATTTATTAGGCGAAGATGATTG	4663350–4663327
VS1065_F_	GAAAATAGATCTCACATGTTCTTTCCTGCGTTATC	pBluescript II
VS1068_R_	GAAAATGGTTACCCTTAATGCGCCGCTACAGG	pBluescript II
VS1085_F_	GATCACTCTAGAAAAGGCACTGCCACAAAGAAAC	4659927–4659948
VS1086_R_	GATCACTCTAGAGTAATGGTTTATCTGCTTCATAG	4664194–4664172
VS1087_F_	GATCACTCTAGAACTGTCTGGTGTTTTTTGCTAG	1547911–1547932
VS1088_R_	GATCACTCTAGACCTCAATGATTAGTCATCCTTG	1549630–1549609

^a^ Nucleotide sequences of primers used in this study were selected from the published genome sequence of *E*. *coli* O157:H7 strain EDL933 with the accession number AE005174.2.

^b^ Location refers to the position of primer sequence in the genome of EDL933.

^c^ Subscripts F and R denote forward and reverse primers, respectively.

### Determination of Congo red binding

Binding of Congo red by bacterial cells was monitored as described previously [33, 41]. Briefly, O157 strains were inoculated on YESCA containing 1.5% noble agar, Congo red (40 μg/ml), coomassie brilliant blue (6.24 μg/ml) and carbenicillin (100 μg/ml). The plates were incubated at 28°C for 24–48 h. The intensity of the red color of bacterial growth produced on this medium was captured by photography.

### Visualization of curli fimbriae by transmission electron microscopy

For determining the presence of curli fimbriae, bacterial cells from 48 h old plates were suspended in 2.5% glutaraldehyde solution, affixed to formvar grids, and stained with phosphotungstic acid. The stained grids were examined by transmission electron microscopy.

### Biofilm quantification

The biofilm formation was determined by previously published procedures [33, 41]. Briefly, bacterial cultures were grown overnight at 37°C (175 rpm) in YESCA broth containing carbenicillin (100 μg/ml). The overnight cultures were diluted 1:10 in YESCA broth, YESCA broth plus 0.1% glucose (YESCA-G), or DMEM containing carbenicillin (100 μg per ml). Two hundred μl of diluted cultures were added to the wells of a 96-well polystyrene plate. After 48 h of incubation at 28°C or 37°C, the culture liquid was aspirated, the wells were rinsed once with PBS (phosphate-buffered saline), and the plate incubated at 80°C for 30 min. The heat-fixed biofilms were stained with 0.1% crystal violet for 30 min and washed 3-times with distilled water. Biofilm-bound crystal violet was eluted in 95% ethanol and absorbance of eluate was determined at 590 nm (A_590_) using a spectrophotometer.

### Determination of bacterial adherence to HEp-2 cells

Adherence assays were performed as described previously [56, 57]. Briefly, 1 x 10^5^ bacterial cells grown in LB broth (100 μg/ml carbenicillin) overnight at 37°C (200 rpm) were added to HEp-2 cells (cultured in RPMI 1640 containing 10% fetal bovine serum) (Life Technologies, Grand Island, NY) in chambered slides (for microscopic detection of adherent bacterial cells) or in a 24-well tissue culture plate (for enumerating adherent bacterial cells) at ten bacteria to one HEp-2 cell. After incubation for 3 h at 37°C in 5% CO_2_, slides and plates were processed as follows. The slides were washed with Dulbecco’s Phosphate-Buffered Saline (DPBS) (Life technologies, Grand Island, NY) stained with toluidine blue, washed again, air dried, and examined for adherent bacteria under a microscope equipped with a camera. The culture media was aspirated from each well of the 24-well plate and saved into a sterile tube. Each well was washed three times with one ml of DPBS and washes pooled with the aspirated culture medium corresponding to that well to recover non-adherent bacterial cells. To recover adherent bacterial cells, washed HEp-2 cells in each well were treated with 150 μl of 1% Triton-X100 for 10 min at room temperature. The HEp-2 cell lysate was mixed with 850 μl of DPBS and homogenized by repeated pipetting. Ten-fold serial dilutions of the pooled culture medium and HEp-2 cell lysates were plated on LB agar (carbenicillin 100 μg/ml) plates and incubated at 37°C to determine the number of non-adherent and adherent bacterial cells, respectively. Non-adherent and adherent bacterial counts were obtained from three independent assays with each assay performed in duplicate wells.

### Detection of EspA and CsgA

Western blotting was used for detection of EspA and CsgA produced by parental, mutant, and complemented mutant strains. For detection of EspA, bacterial cultures were grown at 28°C or 37°C in YESCA broth, YESCA broth supplemented with 0.1% glucose (YESCA-G), or DMEM containing 100 μg per ml of carbenicillin till the OD_600_ reached between 0.8–0.1(about 3.5 h). The cells were harvested by centrifugation (10, 000 x g) for 5 min and the cellular pellet was suspended in 2X SDS-sample buffer (Bio-Rad, Hercules, CA). The detection of CsgA was accomplished by growing bacterial strains in YESCA broth, YESCA-G broth, or DMEM containing carbenicillin (100 μg per ml) for 48 h at 28°C or 37°C. An aliquot of these cultures, standardized to roughly the equivalent of OD_600_, was centrifuged as above and the cellular pellet was acidified with formic acid at one-fifth of the volume of the original culture. Curli fimbriae are highly resistant to sodium dodecyl sulfate (SDS) and other chemical treatments that are commonly employed for preparing protein samples for analysis by SDS-polyacrylamide gel electrophoresis (SDS-PAGE). Therefore, bacterial cells were treated with formic acid to depolymerize curli fimbriae to CsgA, the major constituent protein of curli fimbriae, in order to detect this protein by SDS-PAGE. The acidified samples were kept on ice for 10 min and dried in a miVac Centrifugal Vacuum Concentrator (Genevac, Inc., Stone Ridge, NY). The dried pellet was dissolved in 2X SDS-sample buffer (BioRad, Hercules, CA). The 2X-SDS solubilized samples prepared for EspA and CsgA detection were heated for 10 min in a 95°C water bath, loaded in to the wells of a 4–15% SDS-polyacrylamide gel, and subjected to electrophoresis using a Tetra Cell electrophoresis unit according to the manufacturer’s instructions (BioRad, Hercules, CA). The proteins from the gels were transferred to the nitrocellulose membranes using a mini Trans-Blot unit at 30 V overnight according to the manufacturer’s instructions (BioRad, Hercules, CA). Following transfer, membranes were blocked in iBind solution and placed protein-side down on an iBind Card of the iBind Western System (Thermo Scientific, Grand Island, NY). Recommended volumes and dilutions of primary anti-rabbit EspA or anti-rabbit CsgA antibodies (Pacific Immunology, Ramona, CA), iBind wash buffer, and goat-anti-rabbit horseradish peroxidase-conjugated secondary antibody (KPL, Gaithersburg, MD) were added in the designated slots of the iBind system. As a loading control, primary mouse anti-Dnak antibody (AbCam, United Kingdom) was used for probing bacterial cell lysates to ensure that the 75 kD DnaK protein was present in these lysates at similar levels. Following completion of protein transfer, the membranes were exposed to 3,3',5,5'-Tetramethylbenzidine Peroxidase Substrate (KPL) to facilitate detection of blue-colored protein bands of predicted sizes. The membranes were rinsed in water and photographed using an AlphaImager (ProteinSimple, Wallingford, CT).

### Statistical analyses

A two sample, Students-t test was used to determine the significance of the differences in adherence and biofilm formation between the mutant and the complemented mutants relative to the parental strain. The differences were considered significant at *p* < 0.05.

## Results

### Bacterial growth rates varied in response to growth medium and temperature

**Fig 1:**
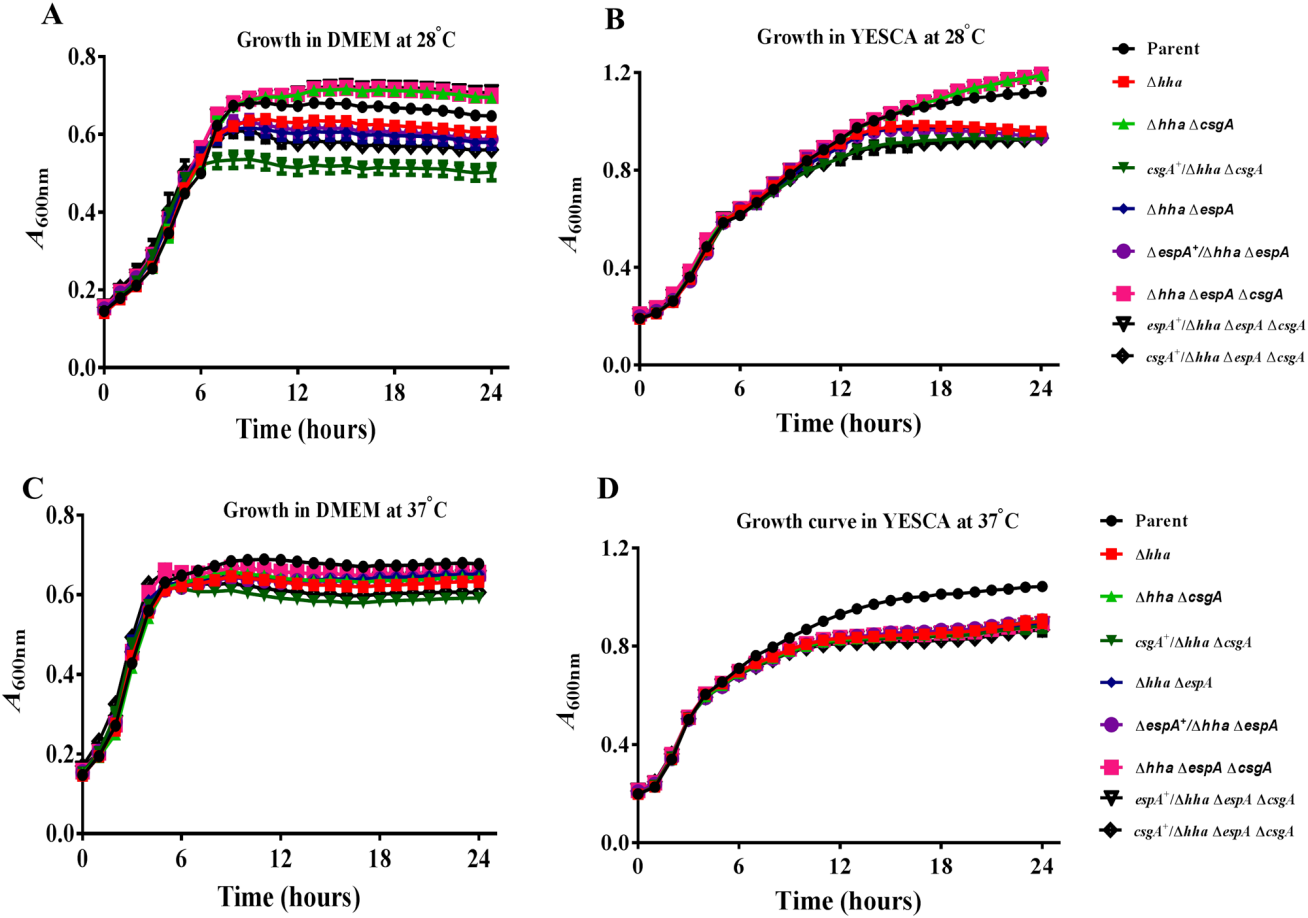
Determination of bacterial growth at 28°C and 37°C in DMEM and YESCA broth. Bacterial strains were grown overnight at 37°C in LB broth containing carbenicillin (100 μg/ml) and diluted 1:1000 in DMEM or YESCA broth containing carbenicillin (100 μg/ml). The diluted samples were incubated at 28°C or 37°C for 24 h and optical density at 600 nm (OD_600_) was recorded every 30 min. Three independent cultures were tested in triplicate, and each point on the growth curve represents an average of nine readings. The generation times were determined by computing the time required for doubling of OD_600_ of the bacterial culture during the exponential phase of growth.

The parental and the eight mutant strains showed identical growth profiles during the exponential phase in DMEM or YESCA broth at 28°C and 37°C (Fig 1). For example, growth in DMEM (Fig 1A) and YESCA broth (Fig 1B) at 28°C resulted in doubling times of about 240 min and 180 min, respectively, for all bacterial strains. Similarly, doubling times of 120 min was observed for the parental and all mutant bacterial strains at 37°C irrespective of their growth in DMEM (Fig 1C) or YESCA broth (Fig 1D). The longer doubling times observed under these experimental conditions were due to the use of an automated growth reader requiring culture volumes of less than one ml and allowing growth increases to occur in smaller increments compared to growth curves generated by growing bacterial strains in large culture vessels with a vigorous shaking. Overall, OD_600_ was markedly lower at 28°C during the stationary phase for bacterial strains expressing greater amounts of CsgA than the parental strain. On the other hand, strains unable to express CsgA due to the deletion of *csgA* have higher OD_600_ compared to the parental strain during the stationary phase at 28°C in DMEM and YESCA broth. All mutant strains had lower OD_600_ during the stationary phase compared to the parental strain at 37°C in DMEM or YESCA broth but the effect was less pronounced at 37°C than that observed at 28°C.

### EspA, but not CsgA, was essential for adherence of O157 to HEp-2 cells

**Fig 2:**
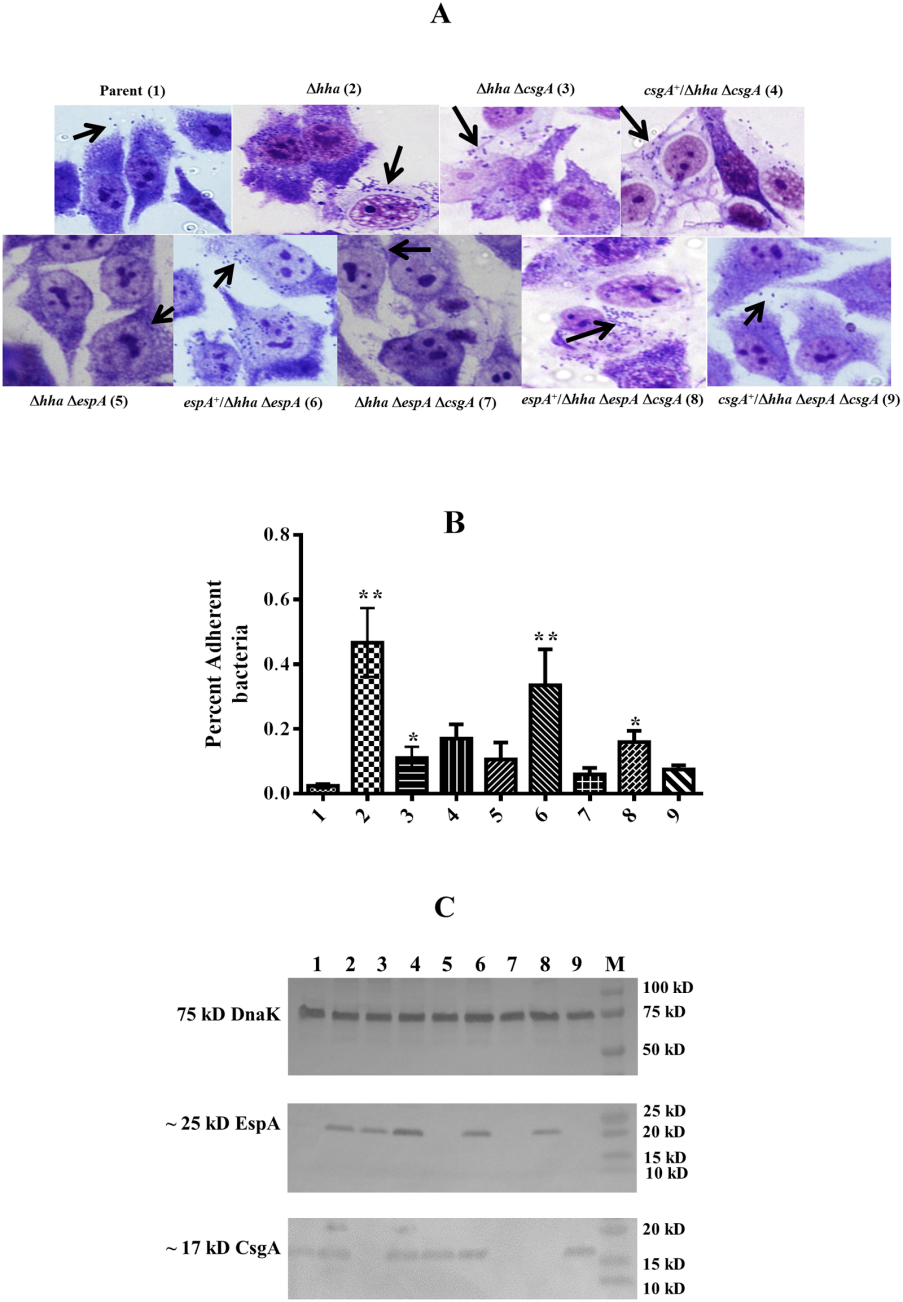
Effects of *espA* and *csgA* deletions on O157 adherence to HEp-2 cells and expression of EspA and CsgA. **(A)** Micrographs showing adherent bacteria on HEp-2 cells (indicated by black arrows) of Parent (1), Δ*hha* (2), Δ*hha* Δ*csgA* (3), *csgA*^+^/Δ*hha* Δ*csgA* (4), Δ*hha* Δ*espA* (5), *espA*^+^/Δ*hha* Δ*espA* (6), Δ*hha* Δ*csgA* Δ*espA* (7), *espA*^+^/Δ*hha* Δ*csgA* Δ*espA* (8), *csgA*^+^/Δ*hha* Δ*csgA* Δ*espA* (9) mutant strains. **(B)** A bar graph showing the percent of adherent bacteria on HEp-2 cells computed by dividing the number of adherent bacterial cells by the sum of non-adherent and adherent bacteria. Bars indicate average of three independent assays and error bars represent standard deviation of 2. The * above the bars indicates *p* < 0.05 when Δ*hha* mutant was compared to the parent and the double and triple mutants were compared to the Δ*hha* mutant strains. **(C)** Western blot analysis of cell lysates (prepared by growing bacterial strains in DMEM at 37°C to an OD_600_ of 0.8 to 1.0) using anti-DnaK (top blot), anti-EspA (middle blot), and anti-CsgA (bottom blot) antibodies. Molecular weights of protein standards (Lane M) are indicated in kD on right side of each blot. The locations of predicted or identified DnaK, EspA, and CsgA proteina are indicated on the left. Lanes numbered 1 through 9 contains cell lysates of strains listed in the same order as in Fig 2A.

Qualitative (microscopic) analysis of bacterial adherence to HEp-2 cells showed that the *hha* mutant adhered to HEp-2 cells in large clusters consisting of several bacterial cells compared to the parental, *hha csgA*, *hha espA*, and *hha espA csgA* mutant strains that adhered to HEp-2 cells mostly as a few individual bacterial cells (Fig 2A). The complementation of *hha espA* and *hha espA csgA* mutants with plasmid pSM706, encoding the *espA* gene, enabled the complemented strains (*espA*^+^/*hha espA*, *espA*^+^/*hha espA csgA*) to adhere to HEp-2 cells as clusters, the adherence pattern resembling to that produced by the *hha* mutant strain (Fig 2A) and indicating the direct requirement of EspA in bacterial adherence to HEp-2 cells. On the other hand, complementation of the *hha espA csgA* mutant with plasmid pSM708, encoding for CsgA, resulted in the adherence phenotype of the complemented strain (*csgA*^+^/*hha espA csgA*) resembling that of the uncomplemented *hha espA csgA* mutant strain showing very few bacterial cells adhering to HEp2-cells (Fig 2A), indicating that CsgA is not essential for the direct adherence of O157 to epithelial cells.

Quantitative analysis of adherence (Fig 2B), which involved removing non-adherent bacterial cells and recovering bacterial cells adhering to HEp-2 cells, showed 19-fold (*p* = 0.002) higher recovery of adherent bacterial cells for the *hha* mutant compared to the parental strain. The percent adherence of the *hha csgA* deletion mutant was 5.5-fold higher (*p* = 0.026) than the parental strain but 4-fold lower (*p* = 0.026) than the *hha* deletion mutant strain. Complementation with a *csgA*-encoding plasmid (pSM708) had no significant effect on the adherence of the *hha csgA* mutant to HEp-2 cells as the percent adherence of the complemented mutant remained 4-fold lower (*p* = 0.026) than the *hha* mutant strain. The adherence of *hha espA* deletion mutant was not significantly (*p* = 0.093) different from the parental strain but was 4.5-fold lower (*p* = 0.0087) than the *hha* mutant strain. However, complementation of the *hha espA* mutant with the *espA*-encoding plasmid (pSM706) enhanced its adherence by 16.5-fold (*p* = 0.002) compared to the parental strain, the level of adherence that was not significantly different (*p* = 0.305) from that of the *hha* mutant strain. The percent adherence of the *hha* mutant deleted of both *espA* and *csgA* (*hha espA csgA*) was not significantly different (*p* = 0.305) from that of the parental strain. Although complementation of *hha espA csgA* mutant with the *espA*-encoding plasmid (pSM706) enhanced its adherence by about 7-fold (*p* = 0.002) relative to the parental strain and by about 3-fold (*p* = 0.032) compared to the uncomplemented *hha espA csgA* mutant strain, the adherence of the complemented (*espA*^+^/*hha espA csgA*) mutant was still 3-fold lower (*p* = 0.02) than the *hha* mutant strain. On the other hand, complementation with the *csgA*-encoding plasmid (pSM708) kept the percent adherence of the complemented (*csgA*^+^/*hha espA csgA*) mutant at a level not significantly (*p* = 0.53) different from the uncomplemented *hha espA csgA* mutant strain, the level of adherence that was still 6-fold lower than the *hha* mutant strain.

Western blot analysis of cell lysates (prepared from bacterial cultures grown to an OD_600_ of 0.8 to 1.0 at 37°C in DMEM-carbenicillin) using anti-EspA and anti-CsgA antibodies showed the presence of 25 kD EspA and 17 kD CsgA bands in lysates of bacterial strains carrying either a chromosomal copy of these two genes or were complemented for the deleted chromosomal copies of these genes with a plasmid-encoded *espA* (pSM706) or *csgA* (pSM708) (Fig 2C). The intensities of the bands corresponding to these two proteins were lowest for the parental strain (Fig 2C) that also showed lowest adherence to HEp-2 cells (Fig 2A and 2B). On the other hand, both EspA and CsgA bands were detected at much higher intensities in the highly adherent *hha* mutant and *hha espA* and *hha espA csgA* mutants that were complemented with plasmid pSM706 (Fig 2C). However, the *hha espA* and *hha espA csgA* mutants, which were unable to produce EspA due to the *espA* deletion (Fig 2C), complemented with plasmid pS708 showed highly reduced adherence compared to the *hha* mutant indicating that EspA is essential for epithelial cell adherence (Fig 2A and 2B). Since 75 kD DnaK protein, used as a loading control, was detected in the whole-cell lysates of all strains at identical levels (Fig 2C), the detection of higher amounts of EspA and CsgA in the *hha* mutant strains harboring either the chromosomal or plasmid-encoded copies of these genes were likely due to the differential expression of these two proteins in these strains.

### Biofilm formation was CsgA-dependent and did not require EspA

**Fig 3:**
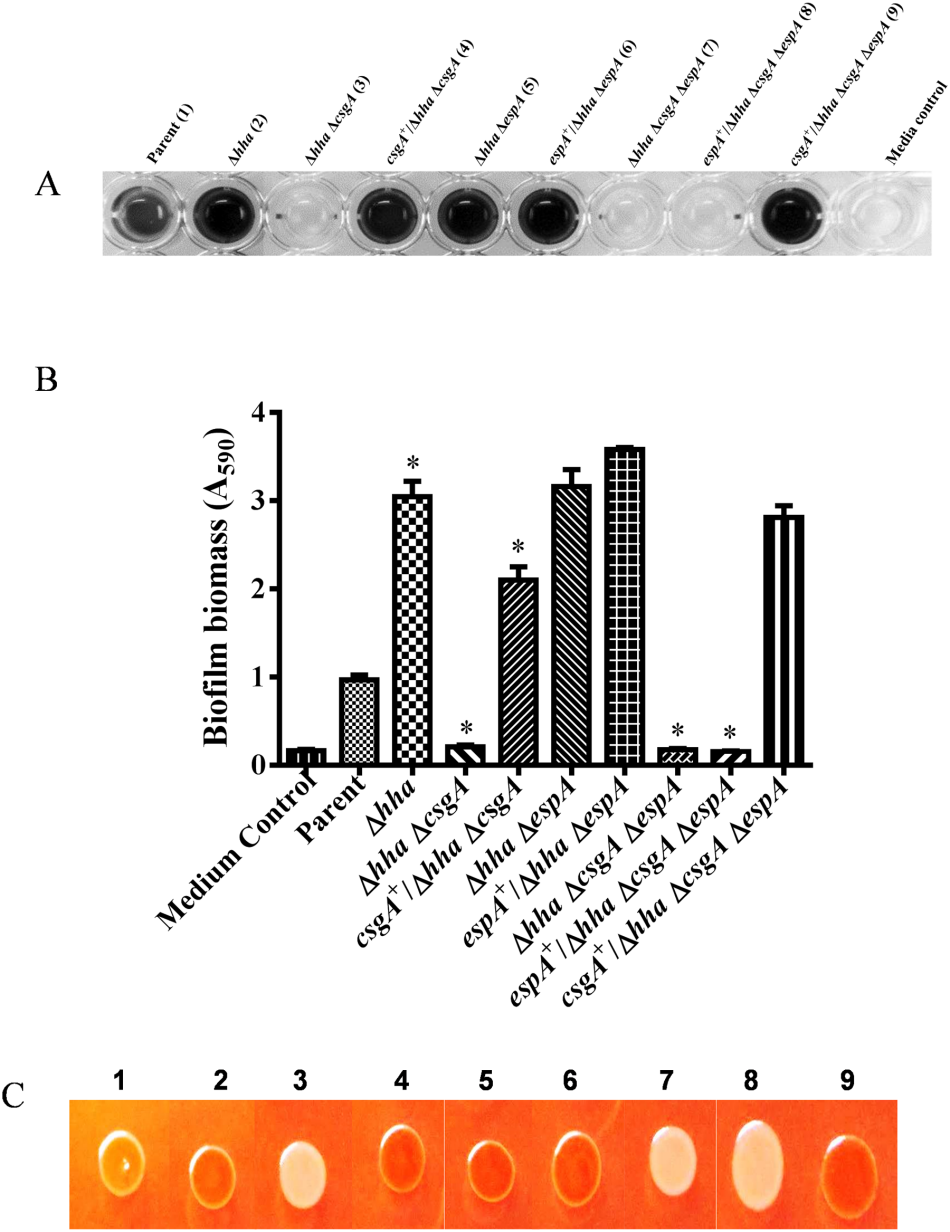
Qualitative and quantitative analysis of biofilm formation in YESCA broth at 28°C. **(A)** Single row of a microtiter plate showing the relative intensity of crystal violet-stained biofilms produced in the wells inoculated with the Parent (1), Δ*hha* (2), Δ*hha* Δ*csgA* (3), *csgA*^+^/Δ*hha* Δ*csgA* (4), Δ*hha* Δ*espA* (5), *espA*^+^/Δ*hha* Δ*espA* (6), Δ*hha* Δ*csgA* Δ*espA* (7), *espA*^+^/Δ*hha* Δ*csgA* Δ*espA* (8), *csgA*^+^/Δ*hha* Δ*csgA* Δ*espA* (9) mutant strains. The last well in the row represents the uninoculated growth medium. **(B)** Bar graph of the amount of biofilms produced by each of the nine strains listed on the X-axis. Bars represent the means of three independent assays and error bars represent standard deviation of 2. The * above the bars indicates *p* < 0.05 when biofilm formation in the mutant strains was compared to the parental strain. **(C)** Color photograph of the bacterial growth produced after 48 h of growth at 28°C on YESCA agar containing Congo red. The numbers 1–9 correspond to strains listed in the same order as in Fig 3A.

Biofilm formation was initially examined after growth of each of the nine bacterial strains in YESCA broth lacking glucose and incubation for 48 h at 28°C. Under these growth conditions, which are normally considered conducive for biofilm formation, the *hha* and *hha espA* mutants produced the highest biofilm biomass. This was indicated by the development of a dark blue color (shown as black color) when the biofilms produced by these two mutants were stained with a crystal violet solution (Fig 3A). On the other hand, the *hha csgA* and *hha csgA espA* mutants were unable to produce detectable amounts of biofilms as the wells of the microtiter plate corresponding to these strains did not stain with crystal violet and appeared similar in coloration to the growth medium control (Fig 3A). When the *hha csgA* and *hha csgA espA* mutants were complemented with *csgA*-encoding plasmid (pSM708) or with the *espA*-encoding plasmid (pSM706), only pSM708 restored biofilm formation to these mutants as indicated by the development of dark blue color with crystal violet staining of the wells inoculated with the complemented (*csgA*^+^/*hha csgA* and *csgA*^+^/*hha csgA espA*) mutant strains (Fig 3A). Quantification (A_590_) of the intensity of the blue color showed that there was a 3-fold (*p* < 0.0001) increase in the production of biofilm by the *hha* and *hha espA* deletion mutants compared to the parental strain (Fig 3B). On the other hand, biofilm production declined by 4.6-fold for the *hha csgA* (*p* < 0.0001) and 5.25-fold for *hha csgA espA* mutants compared to the parental strain (Fig 3B). When compared to the quantity of biofilm biomass produced by the *hha* or *hha espA* mutant, there were15 to 17-fold lower amounts of biofilm produced by *hha csgA* and *hha csgA espA* mutants (Fig 3B). Only plasmid pSM708 but not pSM706 restored biofilm production to the *hha csgA* and *hha cgsA espA* mutants at levels similar to that produced by the *hha* and *hha espA* mutants (Fig 3B).

Since relative ability of O157 isolates to produce biofilms is also correlated with their ability to bind the Congo red dye, we examined the Congo red binding phenotypes of bacterial strains on media containing Congo red. The parental strain that produced very low amounts of biofilm produced lighter red-colored colonies on Congo red medium (Fig 3C). The *hha* and *hha espA* mutants that produced higher amounts of biofilms produced darker red colonies. The *hha csgA* and *hha csgA espA* mutants produced white colonies but upon complementation with pSM708, these strains were able to produce darker red colonies similar to those of *hha* and *hha espA* mutants (Fig 3C).

**Fig 4:**
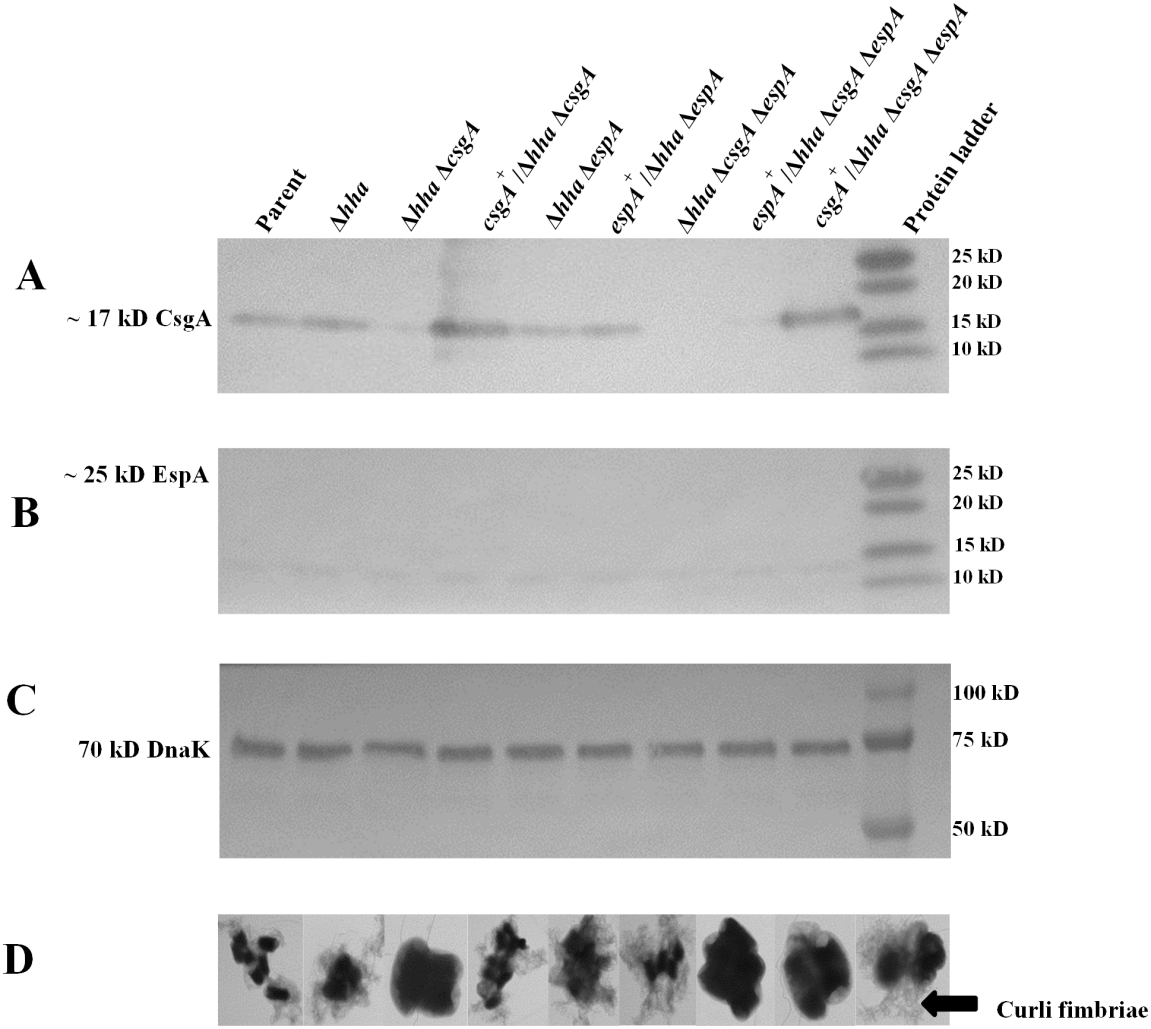
Western blot analysis of cell lysates for detection of EspA and CsgA under growth conditions conducive for biofilm formation. **(A)** Western blot of acid-solubilized cell lysates (prepared from bacterial cells grown in YESCA broth at 28°C for 48 h) probed with anti-CsgA antibody. A predicted protein of 17 kD detected by anti-CsgA antibody in strains carrying the wild-type *csgA* gene is labeled on the left side of the blot. **(B)** Western blot prepared from bacterial lysates and probed with anti-EspA antibody. The location of a predicted EspA protein of 25 kD, not detected in any of the nine strains, is indicated on the left side of the blot. **(C)** Western blot of the loading control protein DnaK generated by probing of bacterial cell lysates with anti-DnaK antibodies. Predicted location of a 75 kD DnaK band detected with anti-DnaK antibody is labeled on the left side of the blot. Molecular weights of protein standards (Lane M) used in the blot are indicated in kD on the right side of each blot. **(D)** Transmission electron micrographs of glutaraldehyde-fixed bacterial cells of each of the nine strains listed on the top of Fig 4A. Dark-stained structures represent bacterial cells and hair like structures present around them are curli fimbriae (indicated by an arrow).

Western blot analysis of formic acid-solubilized bacterial cells (grown in YESCA broth at 28°C for 48 h) using anti-CsgA antibody showed the presence of a ~ 17 kD protein band in the parental, *hha*, and *hha espA* mutant strains that were wild-type for *csgA* (Fig 4A). Similarly, the *hha csgA* and *hha csgA espA* mutant strains that were complemented for *csgA* deletion with plasmid pSM708 also produced a 17 kD band (Fig 4A). However, probing of whole-cell lysates with anti-EspA antibody showed no detectable 25 kD protein bands corresponding to EspA in all nine strains grown at 28°C in YESCA broth at 28°C (Fig 4B). A 75 kD DnaK band was detected in the whole-cell lysates of all strains at similar levels indicating efficient and equivalent extraction of proteins in these lysates (Fig 4C).

In O157, the presence of curli fimbriae at the bacterial cell surface is dependent on the production of CsgA, which is a major structural protein of curli fimbriae. Phenotypic expression of curli fimbriae on bacterial cell surfaces could be determined by negative staining of glutaraldehyde-fixed bacterial cells using a transmission electron microscope [58]. As shown in Fig 4D, with the exception of *hha csgA*, *hha csgA espA*, and *espA*^+^/*hha csgA espA*, all other strains possessed curli fimbriae as determined by electron microscopy of glutaraldehyde-fixed cells. The production of curli fimbriae was restored on *hha csgA* and *hha csgA espA* mutants via complementation with pSM708 (Fig 4D).

### Growth at 28°C in the presence of 0.1% glucose did not induce EspA production but allowed biofilm formation

**Fig 5:**
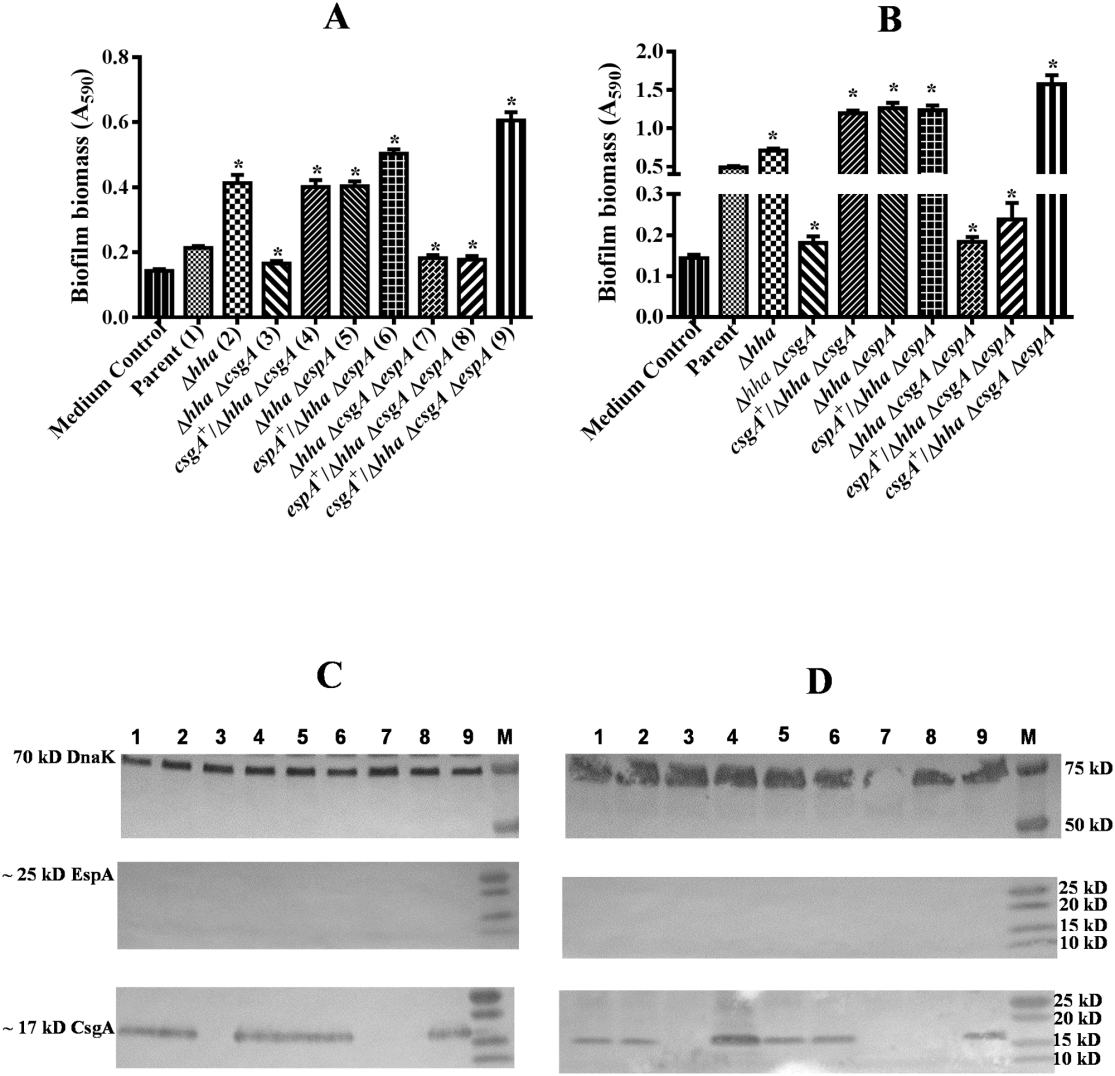
Effect of glucose on EspA and CsgA production and biofilm formation. Bar graphs of biofilms produced in the wells of a microtiter plate after 48 h of growth at 28°C in DMEM **(A)** and YESCA-glucose broth **(B)**. Bars represent means of three independent assays and error bars represent standard deviation of 2. The * above the bars indicates *p* < 0.05 when biofilm formation in the mutant strains was compared to the parental or the *hha* mutant strain. Western blot analysis of cell lysates prepared from bacterial strains grown in DMEM **(C)** and YESCA-glucose **(D)** and probed using anti-DnaK (top blot), anti-EspA (middle blot), and anti-CsgA (bottom blot) antibodies. Molecular weights of protein standards are indicated in kD on the right side of each blot. The locations of predicted or identified DnaK, EspA, and CsgA proteins are indicated on the left. Lanes numbered 1 through 9 contain cell lysates of strains listed in the same order as in Fig 5A and 5B.

Since EspA could not be detected in whole-cell extracts prepared from bacterial strains grown under conditions that maximize biofilm formation (growth at 28°C for 48 h in YESCA broth lacking glucose) (Fig 4B), the role of EspA in biofilm formation could not be discounted unequivocally under these growth conditions. Growth of O157 at 37°C in a minimal essential medium supplemented with glucose has been shown to enhance the production of EspA and other LEE-encoded effector proteins [59]. We were also able to detect EspA in whole-cell lysates of strains harboring a wild-type copy of the *espA* gene after growth at 37°C in DMEM containing 0.1% glucose (Fig 2C). Therefore, we first examined whether growth of bacterial strains at 28°C in DMEM and YESCA broth containing 0.1% glucose (YESCA-G) would induce EspA production and help infer the role of EspA in biofilm formation. The amount of biofilm produced by the *hha* mutant was about 2-fold (*p* < 0.0001) and 1.5 (*p* < 0.0001) higher than the parental strain in DMEM (Fig 5A) and YESCA-G broth (Fig 5B), respectively. Although the magnitude of biofilm biomass produced by the *hha csgA* mutant in DMEM and YESCA-G broth was > 2-fold (*p* < 0.0001) lower than the *hha* mutant strain, complementation of *hha csgA* mutant with *csgA*-encoding plasmid pSM708 increased biofilm formation in the complemented (*csgA*^+^/*hha csgA*) mutant by > 2-fold (*p* < 0.0001), levels similar to those produced by the *hha* mutant strain in DMEM (Fig 5A) and YESCA-G broth (Fig 5B). These results indicated that biofilm formation is dependent on *csgA*. The biofilm production in the *hha espA* and *hha espA* mutant complemented with *espA*-encoding plasmid pSM706 remained at levels (> 2-fold; *p* < 0.0001) as high as in the *hha* mutant strain both in DMEM (Fig 5A) and YESCA-G broth (Fig 5B), indicating that *espA* is not required for biofilm formation. The importance of *csgA* in biofilm formation was further evidenced by the results showing 2 to 3-fold (*p* < 0.0001) reduction in biofilm formation in *hha csgA espA* mutant compared to the *hha* and *hha espA* mutants, and restoration of biofilm formation to levels (> 2-fold higher than the parental strain; *p* < 0.0001) produced by the *hha* and *hha espA* mutants by complementing *hha csgA espA* mutant with plasmid pSM708 but not with pSM706 (Fig 5A and 5B). The maximal amounts of biofilm produced in DMEM and YESCA-G at 28°C were 6- (Fig 5A) and 2-fold (Fig 5B) lower than produced in glucose-free YESCA at 28°C (Fig 3B) indicating that glucose is inhibitory to biofilm formation. Western blotting and probing of formic acid-solubilized bacterial cell pellets, which were collected by centrifugation of cultures grown at 28°C in DMEM (Fig 5C) or YESCA-G (Fig 5D) for 48 h, with anti-CsgA antibody showed the presence of a predicted 17 kD protein band in all strains except the *hha csgA*, *hha csgA espA*, and pSM706-complemented *hha csgA espA* mutant strain. However, no predicted protein band of 25 kD was detected when whole-cell lysates prepared from cultures grown to OD_600_ of 0.8 to 1.0 at 28°C in DMEM (Fig 5C) or YESCA-G (Fig 5D) were probed with anti-EspA antibody indicating that EspA is not required for biofilm formation at 28°C.

### Growth at 37°C in media containing glucose induced EspA production but EspA was not required for biofilm formation

**Fig 6:**
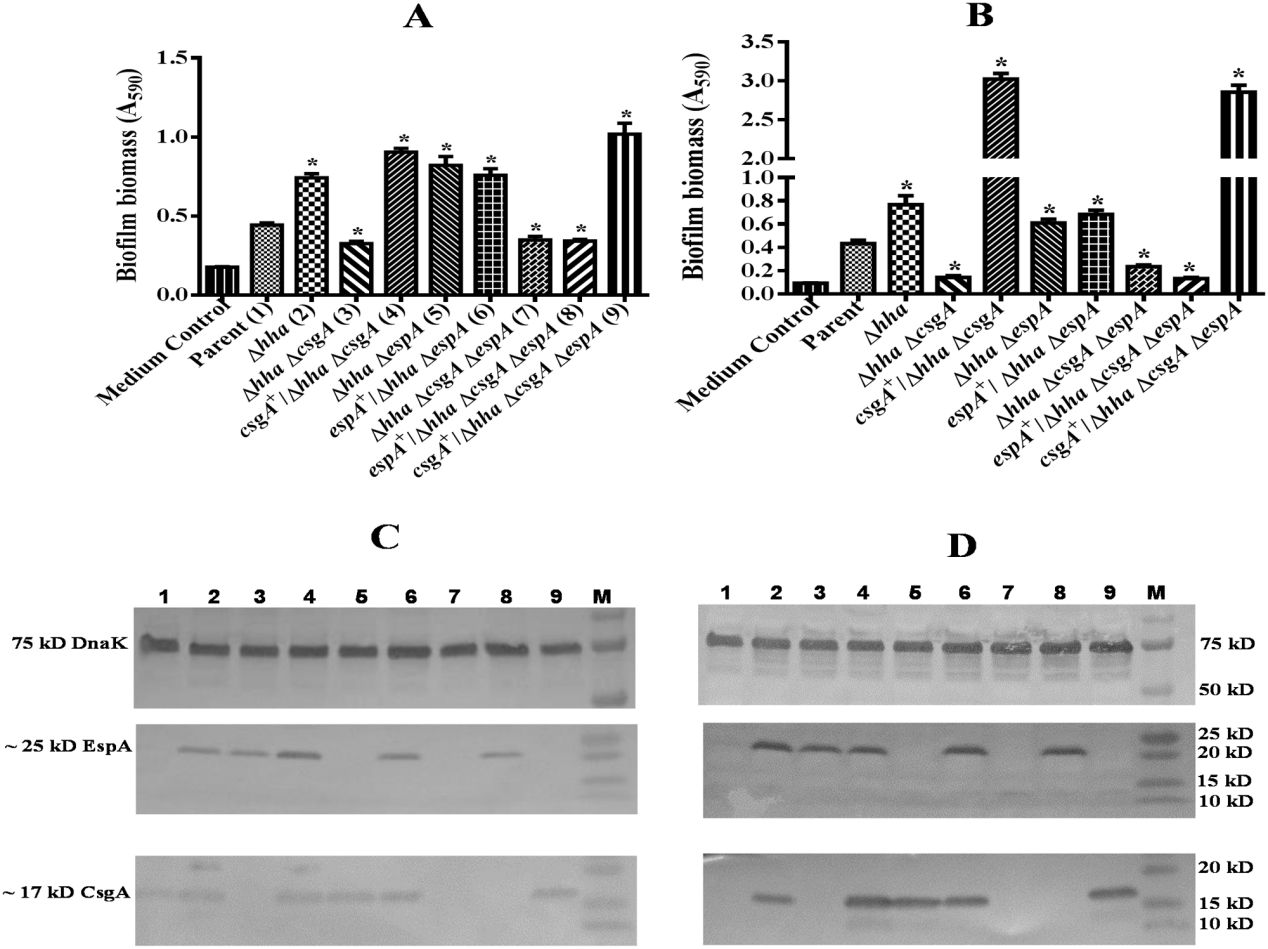
Effect of temperature and glucose on EspA and CsgA production and biofilm formation. Bar graphs of biofilms produced in the wells of a microtiter plate after 48 h of growth at 37°C in DMEM **(A)** and YESCA-glucose broth **(B)**. Bars represent means of three independent assays and error bars represent standard deviation of 2. The * above the bars indicates *p* < 0.05 when biofilm formation in the mutant strains was compared to the parental or the *hha* mutant strain. Western blot analysis of cell lysates prepared from bacterial strains grown in DMEM **(C)** and YESCA-glucose **(D)** and probed using anti-DnaK (top blot), anti-EspA (middle blot), and anti-CsgA (bottom blot) antibodies. Molecular weights of protein standards are indicated in kD on the right side of each blot. The locations of predicted or identified DnaK, EspA, and CsgA proteins are indicated on the left. Lanes numbered 1 through 9 contain cell lysates of strains listed in the same order as in Fig 6A and 6B.

When bacterial strains were grown for 48 h in DMEM and YESCA-G at 37°C, with the exception of the *hha csgA*, *hha csgA espA*, and pSM706-complemented *hha csgA espA* mutant strains, all other strains produced significantly (*p* < 0.05) higher biofilm biomass than the parental strain (Fig 6A and 6B). For example, the magnitude of biofilm produced by the *hha* mutant was about 2-fold (*p* < 0.0001) higher than the parental strain, but biofilm formation in the *hha csgA* mutant was > 2-fold less than the *hha* mutant strain in DMEM (Fig 6A) and YESCA-G broth (Fig 6B). However, complementation of the *hha csgA* mutant with plasmid pSM708 increased biofilm formation in the complemented (*csgA*^+^/*hha csgA*) mutant by > 2-fold (*p* < 0.0001), levels similar to those produced by the *hha* mutant strain in DMEM (Fig 6A) and YESCA-G broth (Fig 6B). The biofilm production in the *hha espA* mutant and *hha espA* mutant complemented with plasmid pSM706 remained at levels ≥ 2-fold (*p* < 0.0001) higher than the parental strain but similar to those produced by the *hha* mutant strain (Fig 6A) and (Fig 6B), indicating that *csgA* is indispensable for biofilm formation. The absolute requirement of *csgA* in biofilm formation was affirmed by results showing ≥ 2-fold (*p* < 0.0001) reduction in biofilm formation in *hha csgA espA* mutant compared to the *hha* and *hha espA* mutants, and restoration of biofilm formation to levels (> 2-fold higher than the parental strain; *p* < 0.0001) similar to those produced by the *hha* mutant by complementing the *hha csgA espA* mutant with plasmid pSM708 but not with pSM706 (Fig 6A and 6B). Western blot analysis using anti-CsgA antibody of formic acid-solubilized bacterial cell pellets, prepared from cultures grown at 37°C in DMEM (Fig 6C) or YESCA-G (Fig 6D), showed the presence of a predicted 17 kD protein band in all strains except for the *hha csgA*, *hha csgA espA*, and pSM706-complemented *hha csgA espA* mutant strains. Similarly, with the exception of the *hha espA*, *hha csgA espA*, and pSM708-complemented *hha csgA espA* mutant strains, all other strains showed the presence of a 25 kD EspA in Western blots of cell lysates prepared from bacterial cultures grown to an OD_600_ of 0.8 to 1.0 at 37°C in DMEM (Fig 6C) or YESCA-G (Fig 6D). These results indicated that the addition of glucose and an incubation temperature of 37°C are critical to induce *espA* expression. Despite production of EspA in DMEM and YESCA-G at 37°C, EspA was dispensable for biofilm formation because complementation of the *hha espA csgA* mutant with plasmid pSM706 failed to restore biofilm formation on this mutant to the levels produced by the *hha* mutant strain.

## Discussion

In the present study, we show the relative importance of two outer membrane filamentous adhesins in adherence of O157 to cultured epithelial cells and abiotic surfaces. The EspA filaments along with other virulence proteins encoded by the LEE pathogenicity island are needed for initial adherence to and subsequent translocation of effector proteins into epithelial cells [15, 16]. Curli fimbriae belong to the amyloid class of proteins and are responsible for bacterial interactions with both biotic and abiotic surfaces and subsequent interbacterial interactions considered important during biofilm formation [42]. We have shown previously that *hha* is a negative transcriptional regulator of LEE-encoded pathogenicity island and operons *csgDEFG* and *csgBAC* encoding genes for biosynthesis of curli fimbriae that are essential for biofilm formation [33, 50]. Thus the *hha* deletion mutant, in which expression of genes encoded by LEE and the two curli operons is highly upregulated, provided an important model system to examine the specific contributions of EspA filaments and curli fimbriae in adherence of O157 to epithelial cells and to polystyrene surfaces during biofilm formation. By deleting *espA* and *csgA*, or both *espA* and *csgA* genes in the *hha* mutant strain, we showed that *espA* and *csgA*, which encodes for EspA filaments and curli fimbriae, respectively, function independently of each other with each having a dedicated function.

It has been shown that EspA filaments are produced by only a fraction of O157 bacterial population through a posttranscriptional mechanism controlled by regulators whose expression are controlled by specific environmental signals and phase-variable events [60]. In agreement with these findings, Western blot analysis of whole-cell lysates with anti-EspA antibody showed very low-levels of EspA production in the parent compared to the isogenic *hha* deletion mutant strain. We also observed a direct correlation between magnitudes of adherence to HEp-2 cells with the levels of EspA production. The *espA* deletion mutant of *hha* showed highly reduced adherence compared to the parental strain whose adherence was several-fold lower than the *hha* mutant strain. Moreover, presence of high levels of curli fimbriae alone was not able to compensate for the loss of adherence of the *hha espA* deletion mutants. These findings clearly demonstrated that increased production of EspA not only enhanced the magnitude of adherence, but it was also a critical element for the adherence of O157 to epithelial cells.

Curli fimbriae are encoded by the *csgA* gene and are highly diverse in their functional activities [3]. Curli fimbriae, because of their amyloid-like properties, are adhesive cell surface structures and have been shown to interact with a variety of animal tissues and abiotic surfaces [31, 32, 43, 61]. One of the best studied functions of curli fimbriae is in biofilm formation but there are only a few studies describing the importance of curli fimbriae in direct interactions with animal epithelial cells [3, 32, 35, 61]. In the current study employing the *hha* mutant producing higher levels of curli fimbriae, we were able to show that contrary to the EspA-dependency of adherence of O157 to epithelial cells, curli fimbriae were dispensable for direct adherence to HEp-2 cells. This inference is supported by the results showing that complementing *hha csgA* and *hha espA csgA* mutants with a *csgA* encoding plasmid (pSM708) did not enhance adherence of the complemented mutants relative to the non-complemented mutant strains.

However, curli might indirectly contribute to the adherence of O157 to epithelial cells by promoting interbacterial interactions as indicated by the reduction in the adherence of the *hha csgA* mutants to HEp-2 cells compared to the *hha* mutant strain. For example, microscopic observations revealed that the *hha* mutant produced an adherent phenotype characterized by the presence of clusters of adherent cells. This would suggests that once a *hha* mutant bacterial cell adheres to an HEp-2 cell using EspA and other LEE-encoded adherence factors, curli fimbriae present on the adherent mutant bacterial cells might interact with nearby non-adherent cells to produce clusters consisting of many cells. These clusters were not produced by the low-level curli-producing parental and the curli-lacking *hha csgA* mutants indicating that increased recovery of *hha* mutant adherent cells compared to the *hha csgA* mutant might be due to the production of clustered adherence phenotype by the *hha* mutant. Interestingly, the *csgA*-encoding plasmid (pSM708) was able to restore both the Congo red binding and biofilm production to the *hha csgA* mutant after 48 h of incubations, but it failed to restore adherence of this mutant to HEp-2 cells at the levels shown by the *hha* mutant. These results suggest that the length of adherence assays that lasted only three hours might not be long enough for this plasmid to produce substantial levels of curli fimbriae needed to promote clustered adherence phenotype. It is also possible that temporal differences in the expression of *csgA* from the *csgA*-encoding plasmid relative to the chromosomal copy of *csgA* in the *hha* mutant might be a limiting factor for the *csgA*-complemented *hha csgA* mutant to achieve high levels of adherence that is typical of the *hha* mutant strain.

The regulation of the expression of *csgA* and other genes required for biogenesis of curli fimbriae is highly complex and triggered by factors, such as low temperature, low osmolarity, and nutrient limitation [3, 40, 62]. We first examined the abilities of *hha* and the *hha* mutant deleted of *espA* or *csgA* or both *espA* and *csgA* to produce biofilms at 28°C in a medium (low osmolarity and lacking glucose as a carbon source) conducive for biogenesis of curli fimbriae [31]. Under these growth conditions, only the *hha csgA* and *hha csgA espA* mutants were unable to produce detectable amounts of biofilms unless complemented with *csgA*-expressing plasmid, confirming that CsgA production is essential for O157 to produce biofilms. Interestingly, CsgA was detectable in cell-lysates of all mutants as long as they harbored the wild-type *csgA*. However, EspA was not detected in lysates of all nine strains tested in this study.

The expression of *espA* and *LEE* is also very complex as numerous transcriptional regulators and environmental conditions tend to modulate their expression [50, 63–70]. For example, the expression of *LEE*, which encodes the *espA* gene, is induced under conditions mimicking the host animal intestinal environment [59]. It has been demonstrated that optimal expression of *LEE* can be induced in minimal essential medium supplemented with glucose and by utilizing a growth temperature of 37°C. Thus, in order to understand the role of EspA in biofilm formation, we used DMEM and the YESCA broth supplemented with glucose to induce *espA* expression at 28°C and 37°C. None of the strains produced EspA in either of the two media at 28°C but incubation at 37°C allowed EspA production in strains carrying a wild-type *espA*. Although substantial amounts of biofilms were produced at 37°C by the *hha* mutant and the *hha* mutants harboring the wild-type copy of the *csgA* gene, biofilm formation occurred independent of the production of EspA.

While low temperature promotes biofilm formation, glucose at a concentration of 0.1% or higher could potential inhibits initial stages or lower the amount of biofilm formation in *E*. *coli* and other enterobacteriacea [71]. The inhibitory effects of glucose on biofilm formation have in part been linked to cAMP and cAMP receptor protein-mediated catabolite repression affecting expression of genes mediating biofilm formation. We did observe about 60% to 75% reduction in biofilm production in DMEM and YESCA containing 0.1% glucose at 28°C and 37°C despite the detection of roughly the similar levels of CsgA under these conditions. The formation of lower amounts of biofilms observed in the presence of glucose could not be attributed to the differences in bacterial growth rates (120 min and 240 min of doubling times at 37°C and 28°C, respectively) because roughly the similar amounts of biofilms were produced in glucose-containing DMEM and YESCA at 28°C or 37°C. Since highest amounts of biofilms were produced at 28°C in YESCA broth lacking glucose (Fig 3), both the absence of a rapidly metabolizable energy source and growth at low temperatures are necessary for the expression of factors conducive for optimal levels of biofilm formation. Nonetheless, the ability to produce even lower amounts of biofilms at 37°C in the presence of glucose could potentially be advantageous to survival and persistence of O157 in the animal host, considering that low levels of glucose are present in the intestinal environment [71–73].

## Conclusions

In this study, we demonstrated that EspA and CsgA contribute independently to tissue adherence and biofilm formation, respectively. We confirm previous findings that EspA production requires bacterial growth at 37°C in the presence of glucose, conditions that attenuate biofilm formation but still allow substantial expression of this adherence phenotype. Although we determined that curli fimbriae have no direct role in O157 adherence to HEp-2 cells, curli fimbriae might indirectly enhance adherence of O157 by promoting interactions between adherent (mediated through EspA) bacterial cells with the non-adherent cells on epithelial cell surfaces. Since curli fimbriae are also able to interact with a variety of host molecules, curli fimbriae could exploit other matrices or cell surface molecules in the animal intestinal environment to promote bacterial adherence and biofilm formation. A high proportion of clinical isolates of uropathogenic *E*. *coli* and sorbitol fermenting Shiga toxigenic *E*. *coli* isolated from patients express curli fimbriae at 37°C and have been shown to interact with cells of the urinary tract system and colonic cells, respectively [49, 61, 74].
